# Overweight/Obesity Increases the Risk of Overt Hepatic Encephalopathy after Transjugular Intrahepatic Portosystemic Shunt in Cirrhotic Patients

**DOI:** 10.3390/jpm13040682

**Published:** 2023-04-19

**Authors:** Lihong Gu, Xiaochun Yin, Yang Cheng, Xixuan Wang, Ming Zhang, Xiaoping Zou, Lei Wang, Yuzheng Zhuge, Feng Zhang

**Affiliations:** Department of Gastroenterology, Affiliated Drum Tower Hospital, Medical School, Nanjing University, Nanjing 210008, Chinafzdndx@126.com (F.Z.)

**Keywords:** liver cirrhosis, overweight/obesity, hepatic encephalopathy, transjugular intrahepatic portosystemic shunt

## Abstract

The purpose of this study was to investigate the effect of body mass index (BMI) on the prevalence of overt hepatic encephalopathy (OHE) after the transjugular intrahepatic portosystemic shunt (TIPS) procedure in decompensated cirrhotic patients. A retrospective observational cohort study of 145 cirrhotic patients receiving TIPS was carried out in our department from 2017 to 2020. The relationships between BMI and clinical outcomes including OHE, as well as risk factors of developing post-TIPS OHE, were analyzed. BMI was categorized as normal weight (18.5 ≤ BMI < 23.0 kg/m^2^), underweight (BMI < 18.5 kg/m^2^), and overweight/obese (BMI ≥ 23.0 kg/m^2^). Among the 145 patients, 52 (35.9%) were overweight/obese and 50 (34%) had post-TIPS OHE. Overweight/obese patients more frequently had OHE compared with normal weight patients (OR: 2.754, 95% CI: 1.236–6.140; *p* = 0.013). Overweight/obesity (*p* = 0.013) and older age (*p* = 0.030) were independent risk factors for post-TIPS OHE according to the logistic regression analysis. Kaplan–Meier curve analysis suggested that overweight/obese patients had the highest cumulative incidence of OHE (log-rank *p* = 0.0118). In conclusion, overweight/obesity and older age may raise the risk of post-TIPS OHE in cirrhotic patients.

## 1. Introduction

Overweight/obesity is a growing epidemic worldwide which is associated with adverse health events and increased mortality [1,2]. Overweight/obesity has been regarded as a hazard for chronic liver disease that can progress to cirrhosis [3,4]. Hepatic encephalopathy (HE) was the most frequent first decompensating event in a prospective study of 1773 participants with nonalcoholic fatty liver disease (NAFLD) [5]. Moreover, overweight/obesity is an independent predictor for decompensation clinically (including variceal hemorrhage, HE, or refractory ascites) in cirrhotic patients [6]. Transjugular intrahepatic portosystemic shunt (TIPS) is an important treatment tool for clinical decompensations in cirrhosis. However, TIPS worsens or precipitates HE [7], which is the main drawback. Few studies have explored the relationship between obesity and the incidence of post-TIPS HE in cirrhotic patients.

HE is a neuropsychiatric syndrome that largely comprises cognitive and fine-motor impairment of varying severity [8]. According to the severity, covert HE is referred to minor or no symptoms but abnormal neuropsychological or neurophysiological examinations. Overt HE (OHE) includes grade II and higher grades according to the West Haven criteria, distinguished by disorientation, somnolence, and a coma-like state [9]. OHE adversely affects the quality of life, which disrupts personality, self-reliance, and daily living capability [10]. Moreover, OHE is connected with short survival in cirrhotic patients [11,12]. 

While the pathophysiology of HE is not entirely understood, ammonia and inflammation are considered two major pathogeneses of HE. The capacity of hepatocytes to eliminate ammonia is impaired in liver cirrhosis, and hyperammonemia ensues, leading to excess ammonia across the blood–brain barrier, the accumulation of intra-astrocytic glutamine, and low-grade cerebral oedema [13,14,15]. Inflammatory factors and hyperammonemia synergistically contribute to astrocytic and neuronal dysfunction, leading to alterations in synaptic plasticity and oscillatory networks; thus, HE-related neurologic symptoms occur [16,17]. However, no direct association between hyperammonemia and the degree of HE was observed [18]. Thus, predicting factors of post-TIPS OHE remain scarce for cirrhotic patients, and more risk factors should be involved.

Taken together, we hypothesized that overweight/obesity might be a predictor for post-TIPS OHE in cirrhotic patients. However, the aspect has not been adequately investigated so far, which was the objective of this study.

## 2. Materials and Methods

### 2.1. Patients

The Ethics Committee of the Affiliated Drum Tower Hospital of Nanjing University Medical School approved the retrospective study, which followed the rules of the 1975 Declaration of Helsinki (protocol #: 2018-276-02). Patients lacking weight and height statistics were excluded from the present study. We retrospectively screened 155 consecutive patients aged 18 to 80 years old with decompensated liver cirrhosis for TIPS due to refractory ascites and gastroesophageal variceal bleeding from January 2017 to December 2020 from our prospectively established database. Written informed consent was acquired from all patients before TIPS. Ten patients were excluded due to hepatocellular carcinoma (HCC). The final sample thus included 145 participants. 

BMI was valued by dividing weight in kilograms by the square of height in meters. All participants were classified as normal weight (18.5 ≤ BMI < 23.0 kg/m^2^), underweight (BMI < 18.5 kg/m^2^), and overweight/obese (BMI ≥ 23.0 kg/m^2^) according to the WHO classification for Asian populations [19].

### 2.2. Transjugular Intrahepatic Portosystemic Shunt Procedure

Employing a transjugular venous approach, the right or middle hepatic vein was catheterized as described previously [20]. The hepatic vein and portal vein were punctured through an intrahepatic tract. Then PPG was assessed during the procedure. A 6–8 mm diameter covered stent (Fluency, Bard, Murray Hill, NY, USA) combined with a bare metal stent (Luminexx, Bard, Murray Hill, NY, USA) or a Viatorr stent (Gore Viatorr, Gore&Associates, Newark, DE, USA) was deployed into the tract to support the parenchymal channel. A balloon catheter was used to dilate the stent. The length of the stent was determined with an opaque marked catheter. PPG was assessed again immediately after stent placement. The residual varices were embolized with coils or tissue-adhesive glue when apparent stomach and esophageal varices existed. Stent patency was evaluated by doppler ultrasonography or TIPS imaging 5 days after the insertion of TIPS.

### 2.3. Follow-Up

Patients were regularly followed through outpatient visits every month for the first 3 months after TIPS and then with an interval of 1–3 months via telephone and/or outpatient clinic until November 2022. During the follow-up, death, liver transplantation, variceal rebleeding, shunt dysfunction, and post-TIPS OHE were recorded. The major endpoint was liver transplantation-free survival. A survival time was valued from the date that TIPS was performed.

During follow-up, the presence of OHE was diagnosed according to signs and symptoms such as mental abnormalities and neurological abnormalities [9]. The detection of asterixis and disorientation was regarded as a symbolic symptom of OHE. Psychometric testing for minimal HE was not conducted.

### 2.4. Statistical Analysis

SPSS 26.0 (IBM Corp, Armonk, New York, NY, USA) was applied for data analysis. Continuous variables were presented as the median (interquartile range, IQR), and categorical variables were presented as the frequency (percentage). The difference in the continuous normal and nonparametric data among the three groups was evaluated via one-way ANOVA analysis and a Kruskal–Wallis test, respectively. The difference in the continuous normal and nonparametric data between the two groups was evaluated using an unpaired t test and Mann–Whitney U test, respectively. The categorical data were evaluated by a Chi-squared test or Fisher’s exact test. Kaplan–Meier curve analysis was applied for survival probability curves, and the comparison was carried out by a log-rank test via Prism 8 (GraphPad Software) and R studio (version 4.2.1) and Survival and Survminer for Kaplan–Meier curves. Univariate logistic regression analysis was used to investigate the relationship between BMI and OHE, and multivariable logistic regression analysis was performed by adjusting for age, gender, and Cr to identify whether BMI was an independent predictor of OHE. Odds ratios (ORs) with 95% CIs were valued. A *p* value less than 0.05 was considered statistically significant.

## 3. Results

### 3.1. Patients’ Baseline Characteristics

**Figure 1:**
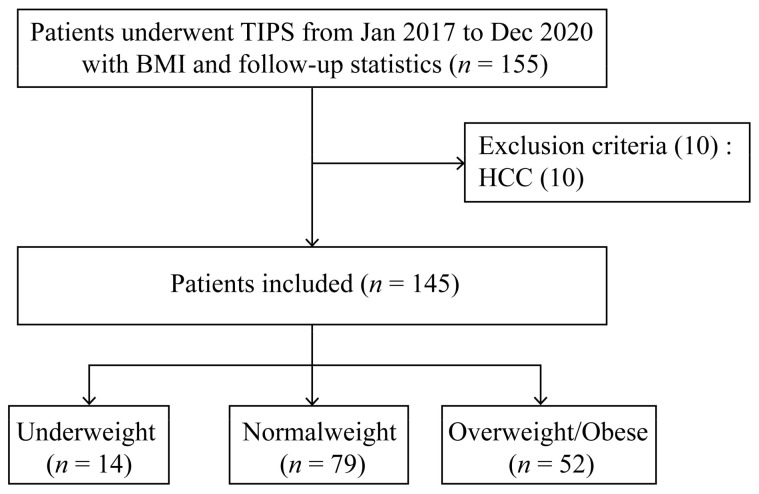
Patient flow chart. BMI, body mass index; HCC, hepatocellular carcinoma; TIPS, transjugular intrahepatic portosystemic shunt.

Overall, we screened 155 cirrhotic patients who underwent the TIPS procedure with BMI and follow-up statistics in Nanjing Drum Tower Hospital. Ten patients were excluded due to the presence of HCC. Finally, a total of 145 participants were included in the present study (Figure 1). The median age of these participants was 58 years, ranging from 50 to 66, with 92 (63.4%) males. Table 1 overviews the patients’ baseline characteristics. Among these patients, 14 (9.7%) were underweight and 52 (35.9%) were overweight/obese. The median BMI was 21.09 (IQR, 19.84–21.97 kg/m^2^) in the normal weight group, 17.75 (IQR, 17.20–18.08 kg/m^2^) in the underweight group, and 24.98 (IQR, 23.88–26.49 kg/m^2^) in the overweight/obese group. Among all 145 patients, 109 (75.2%) received 8 mm stents, and the others received 6 mm stents. According to the BMI subgroup classification, the patients receiving 8 mm stents accounted for 85.7% (12/14) in the underweight group, 70.9% (56/79) in the normal group, and 78.8% (41/52) in the overweight/obese group, respectively. No marked difference was observed among the three groups (*p* = 0.370). The etiologies of cirrhosis involved virus (Hepatitis B or C) and others (alcoholism, primary biliary cirrhosis, autoimmune hepatitis, or schistosomiasis cirrhosis). There was no difference among the three groups according to BMI classification with respect to age, gender, etiology, ascites, diabetes, hypertension, pre-TIPS OHE, the experience of splenectomy surgery, pre-TIPS portal thrombosis, laboratory tests, MELD score, CTP score/grade, stent diameter, or duration of follow-up (*p* > 0.05 for all).

### 3.2. Hemodynamic Index and Clinical Outcomes

Table 2 demonstrates the pre-TIPS and post-TIPS hemodynamic index, NH_3_, and post-TIPS clinical outcomes. The hemodynamic success of TIPS (final PPG ≤ 12 mmHg) was not achieved in 29 patients (20%). The median pre-TIPS PPG was 22 (IQR, 20–27 mmHg), which decreased to 9 (IQR, 6–12 mmHg) after the TIPS procedure. The overweight/obese group had significantly lower pre-TIPS PPG (20, IQR, 18–25 vs. 24, IQR, 20–28; *p* = 0.005) versus the normal weight group. No significant difference in pre-TIPS PPG between the normal weight group and underweight group (25, IQR, 22–27 vs. 24, IQR, 20–28; *p* = 0.622) was observed. No significant difference was observed among the three groups according to BMI classification with respect to post-TIPS hemodynamic index, including the PPG values (*p* = 0.780), the percentage of final PPG ≤ 12 mmHg (*p* = 0.832), the percentage of PPG drop (*p* = 0.548), the percentage of PPG drop ≥ 50% baseline (*p* = 0.250), or the percentage of good response to TIPS (*p* = 0.913).

The median pre-TIPS NH_3_ was 25.50 (IQR, 12.50–40.00 μmol/L), which markedly increased to 33.00 (IQR, 22.00–49.00 μmol/L) after the TIPS procedure (*p* = 0.003). According to the BMI subgroup classification, the NH_3_ levels significantly increased from 25.00 (IQR, 14.00–39.50) to 32.00 (IQR, 18.75–49.25) in the normal weight group after TIPS (*p* = 0.022), but no significant difference was observed between pre-TIPS and post-TIPS NH_3_ levels in underweight or overweight/obese patients (*p* = 0.403 and 0.077, respectively).

**Figure 2:**
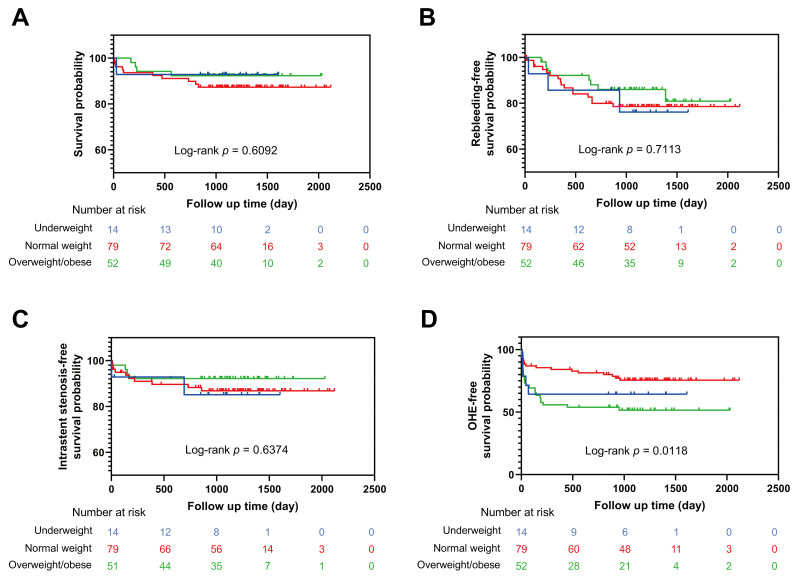
Outcomes after TIPS in patients via the Kaplan–Meier method. (**A**) The cumulative survival probability among the normal weight group, underweight group, and overweight/obese group. (**B**) The cumulative rebleeding-free survival probability among the three groups. (**C**) The cumulative intrastent stenosis-free survival probability among the three groups. (**D**) The cumulative OHE-free survival probability among the three groups. OHE, overt hepatic encephalopathy.

During a median follow-up period of 1224 (IQR, 1044–1443) days, 27/145 (19%) had variceal rebleeding, 17/145 (12%) had intrastent stenosis, and 15/145 (10%) died. Among the 15 death cases, 4 died of hepatic failure, 1 died of lung infections caused by hormones, 1 died of cerebral hemorrhage, 1 died of renal failure, 1 died of cardiac arrest, 3 died of multiple organ failure, 1 died of bleeding, and 3 died of HE. None of the 145 participants underwent liver transplantation. In our department, most patients who underwent liver transplantation had HCC and were excluded from the present cohort. Based on the Kaplan–Meier curve analysis, there were no marked differences in the cumulative survival rate, cumulative rebleeding-free survival rate, and cumulative intrastent stenosis-free survival rate among the normal weight group, underweight group, and overweight/obese group (log-rank *p* = 0.6092, 0.7113, and 0.6374, respectively) (Figure 2A–C).

### 3.3. Relationship between BMI and Post-TIPS OHE

Among 145 patients, 50 (34%) developed OHE after TIPS (Table 2). There was no significant difference regarding the prevalence of post-TIPS OHE between the normal weight group and the underweight group (20, 25% vs. 5, 36%; *p* = 0.514). The overweight/obese group had a markedly higher prevalence of post-TIPS OHE compared with the normal weight group (25, 48% vs. 20, 25%; *p* = 0.007), which was consistent with the outcome of the logistic regression (Table 3). Based on univariable analysis, four variables (age, male, BMI group, and Cr; *p* < 0.1 for all) were included in the multivariable logistic regression analysis; age and overweight/obese were found to be independent predictors of post-TIPS OHE. Older patients had a higher risk of the prevalence of post-TIPS OHE compared with younger ones (OR: 1.041, 95% CI: 1.004–1.080; *p* = 0.030). Overweight/obese patients had a higher hazard of having post-TIPS OHE compared with normal weight patients (OR: 2.754, 95% CI: 1.236–6.140; *p* = 0.013). The Kaplan-Meier curve analysis described that overweight/obese patients had the lowest OHE-free survival probability among the three groups, which means the highest cumulative prevalence of post-TIPS OHE (log-rank *p* = 0.0118) (Figure 2D).

## 4. Discussion

In this study, we investigated the association between BMI and post-TIPS OHE in cirrhotic patients, and the data showed that overweight/obesity was an independent risk factor for OHE. Overweight/obese patients have a higher risk of the prevalence of post-TIPS OHE compared with normal weight patients. The mechanisms underlying the relationship between overweight/obesity and OHE remain unclear, and there are some underlying explanations.

First, in the setting of overweight/obesity, some vital body compositional alterations may occur to promote OHE. Total body adiposity is largely associated with fat deposition within muscle or myosteatosis, which impair the capability of skeletal tissue to metabolize ammonia, resulting in an increased risk of OHE [21,22]. The glutamine synthetase activity of muscle may compensate for the damaged ammonia detoxification in cirrhotic livers, in which case the blood ammonia further rises. Recently, the new concept of sarcopenic obesity identifies overweight/obesity with low skeletal muscle function and mass in patients with NAFLD and diabetes mellitus (DM) [23]. Several pathophysiological mechanisms, such as insulin resistance, oxidative stress, increased proinflammatory cytokines, and decreased physical activity, relate sarcopenia with overweight/obesity [24]. Increasing visceral fat causes inflammation, which contributes to sarcopenia [25]. In contrast, sarcopenia lessens physical activity, resulting in decreased energy expenditure and an increased risk of overweight/obesity, indicating a vicious cycle between overweight/obesity and sarcopenia. The improvement in sarcopenia and myosteatosis as well as subcutaneous adipose tissue has been reported to be associated with the amelioration of cognitive impairment [26]. Thus, overweight/obesity and sarcopenia synergistically promote the risk of OHE. In the present study, there was an increasing tendency of the pre-TIPS NH_3_ levels in the overweight/obese group (29.50, IQR, 16.25–40.50) relative to those in the normal weight (25.00, IQR, 14.00–39.50) or underweight groups (9.00, IQR, 9.00–49.50), suggesting the association between overweight/obesity and OHE. However, no significant difference in pre-TIPS NH_3_ levels was observed among these three groups. The significance might be limited by the sample size. Regarding the post-TIPS NH_3_, there was also an increasing tendency of the NH_3_ levels, the median post-TIPS NH_3_ levels in the underweight (32.50, IQR, 10.50–55.00) and normal weight groups (32.00, IQR, 18.75–49.25) were close, and the levels were higher in the overweight/obese group (38.00, IQR, 25.00–49.00). Though not statistically significant, the data suggested that overweight/obesity might aggravate the increased blood ammonia caused by the TIPS placement. Large-scale studies are required in the future.

Second, overweight/obesity is a well-established risk factor for NAFLD that can progress to cirrhosis. Though obesity and DM are related to higher risks of steatosis, no prior study had separated both risk factors for their relative impact. In a US cohort, liver steatosis is primarily associated with a higher BMI and more modestly associated with DM. Type 2 DM promotes advanced fibrosis among participants who are already overweight and obese [27]. Advanced fibrosis is a well-established predictor of future cirrhosis [28,29], suggesting overweight/obesity itself as a basic risk factor for cirrhosis that can be amplified by DM. Overweight/obesity triggers chronic inflammation and oxidative stress, along with the dysregulation of adipokines secretion, leading to the development of ectopic fat accumulation in the liver [30]. The increasing deposition of triglyceride in the liver alters the metabolism of lipid and glucose, which results in insulin resistance and increases the prevalence of hepatic steatosis [31]. Overweight/obesity may coexist with advanced liver diseases caused by other etiologies. Several studies have demonstrated that overweight/obesity negatively impacts cerebral and cognitive functions [32]. Not only advanced chronic liver diseases but even pre-cirrhotic stages of NAFLD can be associated with brain dysfunction and cognitive decline. Moreover, NAFLD is correlated with low-grade brain tissue inflammation and hypoxia in the presence of overweight/obesity, along with metabolic, cerebrovascular, and behavioral changes, suggesting the early stage of overweight/obesity-related encephalopathy [33].

Overweight/obesity is a well-established predictor for cirrhosis. A population-based study of over 10,000 individuals with a 5-year follow-up revealed that being overweight or obese correlated with an over 50% increased risk for death or hospitalization due to cirrhosis in the absence of previous clinical evidence of liver disease [34]. In addition, a 39-year follow-up study concluded that overweight in late adolescence raises the hazard of developing advanced liver diseases later in life [35]. Furthermore, the risk for decompensation gradually increased in overweight/obese patients with diagnosed cirrhosis compared with those who are of a normal weight [6]. Prior studies have revealed that the connection of the genetic variants with ALT levels and cirrhosis may be amplified by obesity [36,37,38]. In a recent work of multi-trait genome-wide association and gene–environment interaction research, 12 cirrhosis-related genetic variants were employed to generate a polygenic score. Their results showed that elevated alcohol consumption or BMI amplified the relationship between polygenic risk and cirrhosis [39]. Interventions targeting lifestyle factors such as overweight/obesity might ease the inherited predisposition towards cirrhosis. In another study, compared with obese participants, patients at an extreme polygenic risk with normal BMI had a lower hazard of developing advanced liver diseases, suggesting that individuals at a high polygenic risk of cirrhosis may survive from elevated genetic risk via weight loss, including lifestyle changes, bariatric surgery, or pharmacotherapy [36].

One challenge in treating overweight/obesity in cirrhotic patients is that the catabolism in cirrhosis may lead to significant muscle loss in the context of calorie restriction [40]. An RCT study demonstrated that the combined intervention of exercise, diet, and branched-chain amino acids (BCAA) supplementation reduced fat mass and improved muscle mass [41]. An adequate diet is essential for the prevention of neurological pathologies that come from systemic metabolic alterations [42]. Another work of research demonstrated that a 16-week intervention of diet management (reduced energy and moderate protein) and exercise could achieve weight loss and a portal pressure drop in overweight/obese cirrhotic patients [43]. Thus, it is reasonable to believe that BMI is an effective screening index for the development of post-TIPS OHE in cirrhotic patients, and an early tailored diet and exercise intervention for these patients might be beneficial.

Many risk factors have been revealed to be connected with HE. Predisposing risk factors such as MELD score, hyponatremia, serum bilirubin, and albumin and precipitating risk factors such as DM, malnutrition, sarcopenia, and a more advanced age can predict HE [22,44,45]. In recent years, DM has been discovered as a potential risk factor for HE development, including post-TIPS OHE [46]. However, in the present study, the association between DM and post-TIPS OHE was not observed (OR: 1.992, 95% CI: 0.831–4.773; *p* = 0.122). We speculated that this was limited by the small sample size.

We also found that older age was an independent risk factor for post-TIPS OHE (OR: 1.041, 95% CI: 1.004–1.080; *p* = 0.030), which was consistent with the results reported by previous studies [20]. Age-related neurocognitive changes and body compositional changes such as myosteatosis and sarcopenia can both contribute to a high risk for the development of OHE in older age [47]. Age and BMI may interact together for the prevalence of OHE. Aging might aggravate the overweight/obesity-related chronic inflammatory state in cirrhotic livers. An intestinal flora imbalance and constipation, which can trigger OHE, are more prevalent in the elderly.

This study had several limitations. First, this was a single-center study, and a validation cohort was not conducted externally to verify our findings. Second, due to the inherent limitations of retrospective research, BMI data were lacking in some severe patients due to an emergency operation, probably leading to a selection bias. This is because they were usually bedridden when admitted, leading to the loss of height or weight data. Third, neither adipose tissue distribution nor muscle mass were evaluated. Fourth, the study only included patients with OHE, and no neuropsychological test for subclinical HE was carried out, which might underestimate the prevalence of HE.

In conclusion, both overweight/obesity and older age were independently connected with post-TIPS OHE in cirrhotic patients. In overweight/obese cirrhotic patients, the incidence of post-TIPS OHE was markedly higher, so more attention should be paid to the body composition improvement of these patients. In the future, prospective large-scale studies are required to determine whether interventions focusing on weight control or body composition improvement have a preventive effect on OHE in overweight/obese cirrhotic patients.

## Figures and Tables

**Table 1:** Characteristics of patients just before the TIPS procedure.

Variables	Total (*n* = 145)	Underweight (*n* = 14)	Normal Weight (*n* = 79)	Overweight/Obese (*n* = 52)	*p* Value
Age (years)	58 (50–66)	52.5 (47.5–58)	58 (51–66)	60.5 (49.5–66.8)	0.468
Gender (male)	92 (63.4%)	10 (71.4%)	46 (58.2%)	36 (69.2%)	0.357
BMI (kg/m2)	21.95 (20.05–24.04)	17.75 (17.20–18.08)	21.09 (19.84–21.97)	24.98 (23.88–26.49)	<0.001
Etiology (virus/others)	73/72	10/4	38/41	25/27	0.252
Ascites * (no/light/medium/heavy)	10/35/32/67	0/1/2/10	5/18/20/36	5/16/10/21	0.342
Diabetes	25 (17.2%)	2 (14.3%)	11 (13.9%)	12 (23.1%)	0.413
Hypertension	13 (9.0%)	1 (7.1%)	7 (8.9%)	5 (9.6%)	>0.999
Pre-TIPS OHE	1 (0.7%)	0 (0%)	0 (0%)	1 (1.9%)	0.455
Spleen	121 (83.4%)	11 (78.6%)	66 (83.5%)	44 (84.6%)	0.864
Portal thrombosis	53 (36.6%)	4 (28.6%)	29 (36.7%)	20 (38.5%)	0.792
PT (s)	14.1 (13.0–15.1)	14.5 (14.1–15.6)	14.2 (12.7–15.1)	13.8 (13.0–15.0)	0.386
INR	1.24 (1.14–1.34)	1.28 (1.23–1.38)	1.24 (1.11–1.33)	1.22 (1.14–1.34)	0.295
Fib (g/L)	1.7 (1.4–2.2)	1.5 (1.2–1.7)	1.8 (1.5–2.3)	1.7 (1.5–2.3)	0.190
ALT (U/L)	18.80 (13.65–26.50)	16.75 (10.90–26.80)	19.9 (13.8–34.3)	18.5 (13.3–24.9)	0.352
AST (U/L)	25.0 (20.2–35.1)	21.4 (16.3–38.5)	24.9 (20.5–37.0)	27.2 (21.1–31.4)	0.339
TB (μmol/L)	15.5 (10.9–22.8)	15.2 (8.4–21.5)	15.2 (10.3–21.9)	16.2 (11.7–23.7)	0.698
ALB (g/L)	34.20 (31.50–37.05)	33.20 (30.25–37.15)	34.30 (31.60–36.90)	33.95 (31.25–37.38)	0.885
Cr (μmol/L)	59 (50–71)	65 (53–78)	60 (48–71)	58 (51–65)	0.408
WBC (*10^9/L)	2.50 (1.80–4.30)	1.95 (1.48–3.75)	2.45 (1.68–4.33)	2.70 (2.00–4.60)	0.228
PLT (*10^9/L)	65.0 (40.5–112.0)	46.0 (39.8–66.8)	63.0 (40.0–102.0)	76.5 (41.0–124.0)	0.185
MELD score	9 (8–11)	10 (9–12)	9 (8–11)	10 (8–11)	0.527
CTP score	7 (6–8)	7 (6–8)	7 (6–7)	7 (6–8)	0.490
CTP class (A/B/C)	56/88/1	5/9/0	27/52/0	24/27/1	0.333
Patients received 8 mm stents	109 (75.2%)	12 (85.7%)	56 (70.9%)	41 (78.8%)	0.370
Duration of follow-up (day)	1224 (1044–1443)	1099 (923–1404)	1230 (1051–1443)	1200 (1034–1470)	0.597

Data are median (interquartile range) or frequency (percentage). * Ascites: Light: patients usually have abdominal distension; the ascites can only be detected by ultrasound examination, with a depth under 3 cm. Medium: patients usually have moderate and symmetrical abdominal distension, with a depth from 3 to 10 cm. Heavy: patients have significant bloating. Detected by ultrasound; ascites occupy the entire abdominal cavity, with a depth over 10 cm. ALB, albumin; ALT, alanine aminotransferase; AST, aspartate aminotransferase; BMI, body mass index; Cr, creatinine; CTP, Child–Turcotte–Pugh; Fib, fibrinogen; INR, international normalized ratio; MELD, model for end-stage liver disease; OHE, overt hepatic encephalopathy; PLT, platelet count; PT, prothrombin time; TB, total bilirubin; TIPS, transjugular intrahepatic portosystemic shunt; WBC, white blood cell.

**Table 2:** Hemodynamic index and clinical outcomes according to BMI subgroup classification.

	Total (*n* = 145)	Underweight (*n* = 14)	Normal Weight (*n* = 79)	Overweight/Obese (*n* = 52)	*p* Value
**Hemodynamic index**					
Pre-TIPS PPG (mmHg)	22 (20–27)	25 (22–27)	24 (20–28)	20 (18–25)	0.005
Post-TIPS PPG (mmHg)	9 (6–12)	10 (6–12)	9 (5–12)	8 (6–11)	0.780
Final PPG ≤ 12 mmHg	116 (80%)	11 (79%)	62 (78%)	43 (83%)	0.832
Percentage of PPG drop (%)	60 (46–75)	61 (55–77)	61 (50–75)	57 (45–73)	0.548
PPG drop ≥ 50% baseline	104 (72%)	11 (79%)	60 (76%)	33 (63%)	0.250
Good response to TIPS *	121 (83%)	12 (86%)	65 (82%)	44 (85%)	0.913
Pre-TIPS NH_3_ (μmol/L)	25.50 (12.50–40.00)	9.00 (9.00–49.50)	25.00 (14.00–39.50)	29.50 (16.25–40.50)	0.483
Post-TIPS NH_3_ (μmol/L)	33.00 (22.00–49.00)	32.50 (10.50–55.00)	32.00 (18.75–49.25)	38.00 (25.00–49.00)	0.537
**Outcomes**					
Variceal rebleeding	27 (19%)	3 (21%)	16 (20%)	8 (15%)	0.752
Intrastent stenosis	17 (12%)	2 (14%)	10 (13%)	5 (10%)	0.827
OHE	50 (34%)	5 (36%)	20 (25%)	25 (48%)	0.027
Death	15 (10%)	1 (7%)	10 (13%)	4 (8%)	0.605

Data are the median (interquartile range) or frequency (percentage). * Good response to TIPS, 50% drop in PPG or to values ≤12 mmHg. OHE, overt hepatic encephalopathy; PPG, portal pressure gradient; TIPS, transjugular intrahepatic portosystemic shunt.

**Table 3:** Logistic regression analysis of the risk factors for post-TIPS OHE.

	Univariable Analysis		Multivariable Analysis	
	OR (95% CI)	*p* Value	OR (95% CI)	*p* Value
Age	1.040 (1.006–1.075)	0.020	1.041 (1.004–1.080)	0.030
Male	2.070 (0.976–4.390)	0.058	1.859 (0.797–4.337)	0.151
BMI				
Normal weight				
Underweight	1.639 (0.491–5.469)	0.422	1.749 (0.485–6.301)	0.393
Overweight/obese	2.731 (1.298–5.746)	0.008	2.754 (1.236–6.140)	0.013
Etiology (virus)	1.414 (0.710–2.815)	0.324		
Diabetes	1.992 (0.831–4.773)	0.122		
Hypertension	1.208 (0.374–3.908)	0.752		
Spleen	1.063 (0.421–2.688)	0.897		
Portal thrombosis	0.964 (0.473–1.966)	0.920		
PT (s)	0.902 (0.726–1.120)	0.350		
INR	0.291 (0.030–2.796)	0.285		
Fib (g/L)	1.331 (0.739–2.398)	0.341		
TB (μmmol/L)	1.001 (0.972–1.032)	0.922		
ALB (g/L)	0.993 (0.912–1.082)	0.878		
Cr (μmol/L)	1.016 (1.001–1.031)	0.033	1.014 (0.998–1.029)	0.079
WBC (*10^9/L)	1.100 (0.947–1.277)	0.213		
PLT (*10^9/L)	1.001 (0.997–1.005)	0.777		
MELD score	1.075 (0.944–1.224)	0.277		
CTP score	1.090 (0.810–1.466)	0.570		
Ascites				
No				
Light	2.667 (0.492–14.461)	0.256		
Medium	2.095 (0.378–11.615)	0.397		
Heavy	2.091 (0.410–10.666)	0.375		

ALB, albumin; BMI, body mass index; CI, confidence interval; Cr, creatinine; CTP, Child–Turcotte–Pugh; Fib, fibrinogen; INR, international normalized ratio; MELD, model for end-stage liver disease; OR, odds ratio; PLT, platelet count; PT, prothrombin time; TB, total bilirubin; WBC, white blood cell.

## Data Availability

The information and data of the study population were extracted from the Hospital Information System. The datasets are not publicly available because the individual privacy of the participants should be protected. Data are available from the corresponding author upon reasonable request.
